# Knockdown of lncRNA NEAT1 suppresses hypoxia-induced migration, invasion and glycolysis in anaplastic thyroid carcinoma cells through regulation of miR-206 and miR-599

**DOI:** 10.1186/s12935-020-01222-x

**Published:** 2020-04-23

**Authors:** Xiangrong Tan, Peng Wang, Jianlin Lou, Jiazheng Zhao

**Affiliations:** 1 Institute of Cancer and Basic Medicine (ICBM), Chinese Academy of Sciences, Hangzhou, Zhejiang China; 2 Department of Head and Neck Surgery, Cancer Hospital of the University of Chinese Academy of Sciences, Hangzhou, Zhejiang China; 3 Department of Head and Neck Surgery, Zhejiang Cancer Hospital, No.1, Banshan East Road, Hangzhou, 310000 Zhejiang China

**Keywords:** ATC, Hypoxia, NEAT1, miR-206, miR-599

## Abstract

**Background:**

Anaplastic thyroid carcinoma (ATC) is one of the most aggressive and lethal malignancies. Long non-coding RNAs (lncRNAs) are being found to play crucial roles in ATC progression. Herein, we focused on the role of nuclear paraspeckle assembly transcript 1 (NEAT1) on ATC progression under hypoxia and underlying mechanisms governing it.

**Methods:**

The expression levels of NEAT1, miR-206 and miR-599 were assessed by quantitative real-time polymerase chain reaction (qRT-PCR). Cell migration and invasion abilities were detected using transwell assays. Glucose consumption and lactate production were determined using a corresponding commercial assay kit. Western blot was performed to evaluate the level of hexokinase 2 (HK2). The targeted interplays between NEAT1 and miR-206 or miR-599 were confirmed by dual-luciferase reporter and RNA immunoprecipitation (RIP) assays. Xenograft model was established to observe the effect of NEAT1 on tumor growth in vivo.

**Results:**

Our data indicated that NEAT1 was highly expressed in ATC tissues and cells, and hypoxia induced NEAT1 expression in ATC cells. NEAT1 depletion repressed ATC cell migration, invasion and glycolysis under hypoxia. Mechanistically, NEAT1 acted as a molecular sponge of miR-206 and miR-599. Moreover, the repressive effects of NEAT1 knockdown on ATC cell migration, invasion and glycolysis under hypoxia were mediated by miR-206 or miR-599. Additionally, NEAT1 knockdown weakened tumor growth in vivo.

**Conclusion:**

In conclusion, our study suggested that a low NEAT1 expression suppressed the migration, invasion, and glycolysis in ATC cells under hypoxia at least partially through modulating miR-206 and miR-599, providing new therapeutic strategies for ATC treatment.

## Highlights

1. NEAT1 depletion repressed ATC cell migration, invasion, and glycolysis under hypoxia.
2. NEAT1 acted as a molecular sponge of miR-206 and miR-599.
3. NEAT1 knockdown suppressed the migration, invasion and glycolysis in ATC cells under hypoxia partially by up-regulating miR-206 and miR-599.

## Background

Anaplastic thyroid carcinoma (ATC), accounting for only 1–2% of all thyroid cancers, is one of the most aggressive and lethal malignancies in humans [1]. Despite rare, 14–39% of deaths related to thyroid carcinoma are attributable to ATC [2]. The incidence rate of ATC has increased from 1973 to 2014, and the mean length of follow-up was 14 months in USA [3, 4]. ATC is characterized by the amass of several oncogenic alterations, and emerging evidence has suggested that the increase of oncogenic alterations contributes to the increased aggressiveness level [1]. Although the developments of treatment methods have provided the survival rate, the prognosis of ATC patients remains very poor [5]. Hypoxia, a vital feature of locally advanced solid tumors, contributes to cancer cell malignant progression, such as altered metabolism, invasiveness, and metastasis [6, 7]. Increased glycolysis is responsible for cancer metastasis through enhancing tumor cell migration and invasion at the circumstances of hypoxia [8]. Therefore, it is very imperative to investigate the novel mechanisms underlying ATC progression under hypoxia.

Long non-coding RNAs (lncRNAs) are an endogenous class of RNA molecules of more than 200 nucleotides that are involved in numerous biological processes [9]. Recently, it has become increasingly clear that the dysregulation of lncRNAs plays a crucial role in ATC progression [10, 11]. Nuclear paraspeckle assembly transcript 1 (NEAT1), transcribed from the endocrine neoplasia type 1 locus on chromosome 11, has been discovered as essential regulators of oncogenesis in multiple human tumors, such as prostate cancer, breast cancer and hepatocellular carcinoma [12–14]. Previous documents had also reported that NEAT1 was up-regulated in papillary thyroid cancer (PTC) tissues and cells, and its deficiency repressed PTC progression through the inhibition of cell proliferation, migration, and invasion [15, 16]. Moreover, recent research uncovered that NEAT1 was highly expressed in ATC tissues and cells, and a low high of NEAT1 sensitized ATC cell to cisplatin [17]. Herein, in this study, we focused on the role of NEAT1 on ATC cell migration, invasion, and glycolysis under hypoxia and underlying mechanisms governing it.

In recent years, the competing endogenous RNA (ceRNA) hypothesis suggests a novel regulatory circuitry in which lncRNAs can function as molecular sponges of specific microRNAs (miRNAs) [18]. Emerging evidence has shown that NEAT1 could exert carcinogenic activity in human cancers by acting as ceRNAs of several miRNAs, such as miR-21 and miR-1224 [13, 19]. Previous works had manifested that miR-206 and miR-599 served as a tumor suppressive role in ATC progression by repressing ATC cell migration and metastasis [20, 21]. Interestingly, the putative complementary sites between NEAT1 and miR-206 or miR-599 predicted by starBase v.3 software prompted us to examine them as potential molecular mediators of NEAT1 in ATC progression under hypoxia.

In this study, we firstly observed the role of NEAT1 knockdown on ATC cell migration, invasion, and glycolysis under hypoxia exposure. Consequently, we explored the interplays of NEAT1 and miR-206 or miR-599 in ATC progression under hypoxia.

## Materials and methods

### Clinical specimens and cell culture

50 clinical specimens, including 25 malignant tissues and 25 adjacent non-malignant tissues were obtained from ATC patients who underwent surgery at Institute of Cancer and Basic Medicine (ICBM), Chinese Academy of Sciences, with written informed consent. All specimens were stored in a freezer at − 80 °C before use. These clinical specimen cohorts were used for this study approved by the Institution Ethics Committee of Institute of Cancer and Basic Medicine (ICBM), Chinese Academy of Sciences.

SW1736 and KAT-18 ATC cell lines and Nthy-ori3-1 normal thyroid cell line were bought from BeNa Culture Collection (Beijing, China) and grown in Dulbecco’s Modified Eagle’s Medium (DMEM, Gibco, Paisley, UK) plus 10% fetal bovine serum (FBS, Gibco), 1% antibiotics (Gibco) at 37 °C in a humidified incubator set at 5% CO_2_. For hypoxia exposure, cells were placed in a 1% O_2_, 5% CO_2_, and 94% N_2_ gas mixture in an incubator.

### Oligonucleotide transfection

SW1736 and KAT-18 cells were maintained in 96-well plates in grown medium to about 70% confluence, and fresh serum-free medium was substituted. For NEAT1 depletion, 30 nM of small interfering RNA (siRNA) targeting NEAT1 (si-NEAT1, GenePharma, Shanghai, China) was transfected into the two cells, and non-targeted siRNA (si-NC, GenePharma) was used as the negative group. For miRNAs overexpression, cells were introduced with 50 nM of modified miR-206 mimic (GenePharma), miR-599 mimic (GenePharma), or a scrambled oligonucleotide sequence (miR-NC mimic, GenePharma). The silencing of miRNAs was carried out using 50 nM of synthetic miR-206 inhibitor (anti-miR-206, GenePharma), miR-599 inhibitor (anti-miR-599, GenePharma), with a non-targeted random sequence (anti-miR-NC, GenePharma) as the negative control. The commercially available Lipofectamine 3000 transfection reagent (Invitrogen, Breda, Netherlands) was employed for each transfection, referring to the protocols of manufacturers.

### Quantitative real-time polymerase chain reaction (qRT-PCR)

SW1736 and KAT-18 cells in 96-well plates were allowed to reach approximately 70% confluence and then exposed to hypoxic conditions, or transfected with the indicated oligonucleotides before hypoxia exposure. The TRIzol reagent (Invitrogen) was used to isolate total RNA from tissue specimens and treated cells. cDNA synthesis was implemented using the High Capacity cDNA Synthesis kit (Applied Biosystems, Rotkreuz, Switzerland) for NEAT1 and TaqMan™ Advanced miRNA cDNA Synthesis kit (Applied Biosystems) for miR-206 and miR-599. qRT-PCR was performed on an ABI 7500HT instrument (Applied Biosystems) with SYBR Premix ExTaq mix (TaKaRa, Dalian, China). Relative expression of NEAT1, miR-206, and miR-599 were normalized according to the internal control of U6 snRNA or glyceraldehyde-3-phosphate dehydrogenase (GAPDH) and calculated by the 2^−ΔΔCt^ method. Primers used for PCR were listed as: NEAT1 (Forward, 5′-TGGCTAGCTCAGGGCTTCAG-3′; Reverse, 5′-TCTCCTTGCCAAGCTTCCTT-3′); GAPDH (Forward, 5′-GAATGGGCAGCCGTTAGGAA-3′; Reverse, 5′-AAAAGCATCACCCGGAGGAG-3′); miR-206 (Forward, 5′-TGGAATGTAAGGAAGTG-3′; Reverse, 5′-CAGTGCGTGTCGTGGAGT-3′); miR-599 (Forward, 5′-GUUGUGUCAGUUUAUCAAAC-3′; Reverse, 5′-CTCCATATCGCACTTTAATCTCTAACT-3′); U6 snRNA (Forward, 5′-CTCGCTTCGGCAGCACA-3′; Reverse, 5′-AACGCTTCACGAATTTGCGT-3′).

### Transwell migration and invasion assay

Migration and invasion of SW1736 and KAT-18 cells after hypoxia exposure or various transfections were investigated using a 24-transwell chamber (Corning, Flintshire, UK) and Matrigel-precoated chamber, respectively. Briefly, cells (5 × 10^5^) were maintained in the upper chamber in serum-free medium, and DMEM media plus 10% FBS was placed in the lower chamber. 24 h later, the penetrated cells through the membrane filter were fixed, stained with 0.1% crystal violet. The number of migrated or invaded cells was counted under a 200× magnification microscope (Leica, Wetzlar, Germany) at five random fields.

### Measurement of glucose consumption and lactate production

SW1736 and KAT-18 cells were treated as described above, and then the supernatant of culture medium was collected for the detection of glucose and lactate concentrations. Glucose consumption and lactate production were determined using the Glucose Colorimetric Assay kit (Biovision, Wehrheim, Germany) and Lactate Assay kit (Biovision), respectively, according to the instructions of producers. The relative concentration was normalized on the basis of total protein amounts.

### Western blot for hexokinase 2 (HK2)

Treated cells were homogenized in RIPA lysis buffer (Thermo Fisher Scientific, Melbourne, Australia) containing protease and phosphatase inhibitors (Sigma-Aldrich, Tokyo, China). Cell lysates (50 µg) were resolved on an 8% SDS polyacrylamide gel, and proteins were transferred to nitrocellulose membranes (Amersham, Buckinghamshire, UK). After being blocked with 4% nonfat milk, the membranes were probed with anti-HK2 antibody (ab209847, Abcam, Cambridge, UK) at a dilution of 1:1000 and anti-β-Actin antibody (ab179467, Abcam) at a dilution of 1:5000 overnight at 4 °C. After that, the membranes were stained with horseradish peroxidase-conjugated goat anti-rabbit IgG (ab6721, Abcam) at a dilution of 1:10,000. The immuno-detected proteins were visualized by chemiluminescence (Amersham) and analyzed with BioMax Light Film (Carestream Health Inc., Tokyo, Japan).

### Bioinformatics and dual-luciferase reporter assay

The targeted miRNAs of NEAT1 were predicted using starBase v.3 software at http://starbase.sysu.edu.cn/. The partial sequences of NEAT1 harboring the complementary sequence for miR-206 or miR-599 were cloned into the dual luciferase psiCHECK2 vector (Promega, Southampton, UK) to construct NEAT1 wild-type reporter (WT-NEAT1 or WT-NEAT1′). Site-directed mutants (MUT-NEAT1 or MUT-NEAT1′) of the target region were generated using the Site-Directed Mutagenesis kit (Yeasen, Shanghai, China). Each reporter construct was cotransfected into SW1736 and KAT-18 cells and miR-206 mimic, miR-599 mimic, or miR-NC mimic. Luciferase activity was detected after 48 h transfection using the Dual-luciferase Reporter Assay System (Promega).

### RNA immunoprecipitation (RIP) assay

SW1736 and KAT-18 cells were transfected with miR-206 mimic, miR-599 mimic, or miR-NC mimic, and then cells were harvested after 48 h. Cell lysates were prepared with RIPA lysis buffer and incubated with anti-Argonaute 2 (anti-Ago2, ab32381, Abcam) or IgG-coupled protein A/G beads for 3–5 h. Beads were washed three times with phosphate-buffered saline (PBS), and ultimately total RNA was obtained and subjected to qRT-PCR assay for NEAT1 enrichment.

### Lentiviral vector transduction

Lentiviral vectors harboring short hairpin RNA (shRNA) targeting NEAT1 (sh-NEAT1) were synthesized by Hanbio (Shanghai, China), and non-targeted shRNA-expressing lentiviral vectors (sh-NC) were used as the negative control. SW1736 cells were infected by the constructed lentiviruses in DMEM medium supplemented with 8 µg/mL polybrene. 24 h after transduction, the cells with positive infection were selected using puromycin at a final concentration of 1 µg/mL.

### Xenograft model assays

For in vivo assays, we used 6-week-old male BALB/c nude mice (Henan Research Center of Laboratory Animal, Zhengzhou, China) to establish xenograft model, after obtaining approval from Animal Ethical Committee of Institute of Cancer and Basic Medicine (ICBM), Chinese Academy of Sciences. All experimental procedures were implemented in accordance with the National Standard of the Care and Use of Laboratory Animals. About 5.0 × 10^6^ SW1736 cells stably transduced with sh-NC or sh-NEAT1 were subcutaneously implanted into nude mice (n = 9). One week later, tumor volume was measured with a caliper every week. In the end, all mice were sacrificed, and tumor tissues were removed for weight and qRT-PCR assay.

### Statistical analysis

Each data group represented 3 biological replicates × 3 technical replicates, and all data were evaluated as mean ± standard deviation (S.D.). Differences between two groups were analyzed using a two-tailed Student’s *t*-test, and differences between multiple groups were determined using one-way analysis of variance (ANOVA) followed by post hoc test, which was performed by SPSS v.20.0 software (SPSS Inc., Chicago, IL, USA). Correlations between NEAT1 level and miR-206 or miR-599 expression in ATC tissue specimens were assessed by using the Spearman test. Statistical significance was set up to *P* < 0.05 in each test.

## Results

### Hypoxia induced NEAT1 expression in ATC cells

**Fig. 1:**
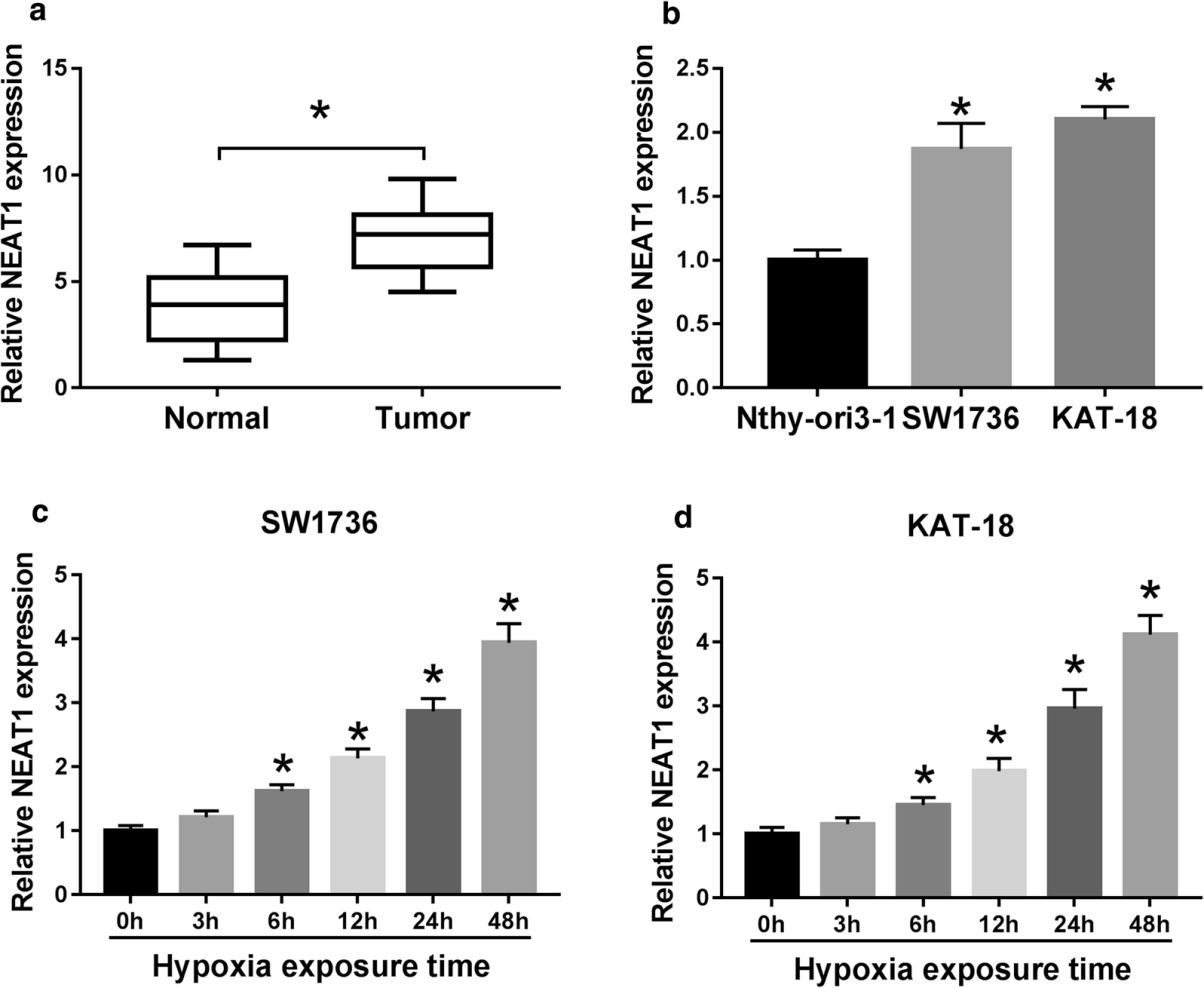
NEAT1 was highly expressed in ATC cells under hypoxic conditions. NEAT1 expression by qRT-PCR in 25 pairs ATC tissues and adjacent normal tissues (**a**), Nthy-ori3-1, SW1736 and KAT-18 cells (**b**). **c, d** SW1736 and KAT-18 cells were exposed to 1% O_2_ for various time points (0, 3, 6, 12, 24 and 48 h), followed by the measurement of NEAT1 level by qRT-PCR. *P < 0.05

Firstly, we determined the expression pattern of NEAT1 in ATC tissues and cell lines (SW1736 and KAT-18). As demonstrated by qRT-PCR, NEAT1 expression was significantly increased in ATC tissues (6.98 ± 0.30) compared with the corresponding normal tissues (3.78 ± 0.32) (*P* < 0.001, Fig. 1a). In parallel, NEAT1 expression was higher in ATC cells (1.87 ± 0.20 in SW1736 cells and 2.11 ± 0.10 in KAT-18 cells) than that of control (1.01 ± 0.08) (*P* < 0.001, Fig. 1b). Then, we detected NEAT1 expression in SW1736 and KAT-18 cells after hypoxia exposure. In contrast to the negative group, hypoxic stress triggered a significant up-regulation of NEAT1 expression in a time-dependent manner in the two ATC cells (*P* < 0.05, *P* < 0.01, *P* < 0.001, Fig. 1c, d). These data together indicated that NEAT1 was up-regulated in ATC tissues and cells and hypoxia-treated ATC cells.

### Knockdown of NEAT1 relieved hypoxia-induced migration, invasion and glycolysis in ATC cells

**Fig. 2:**
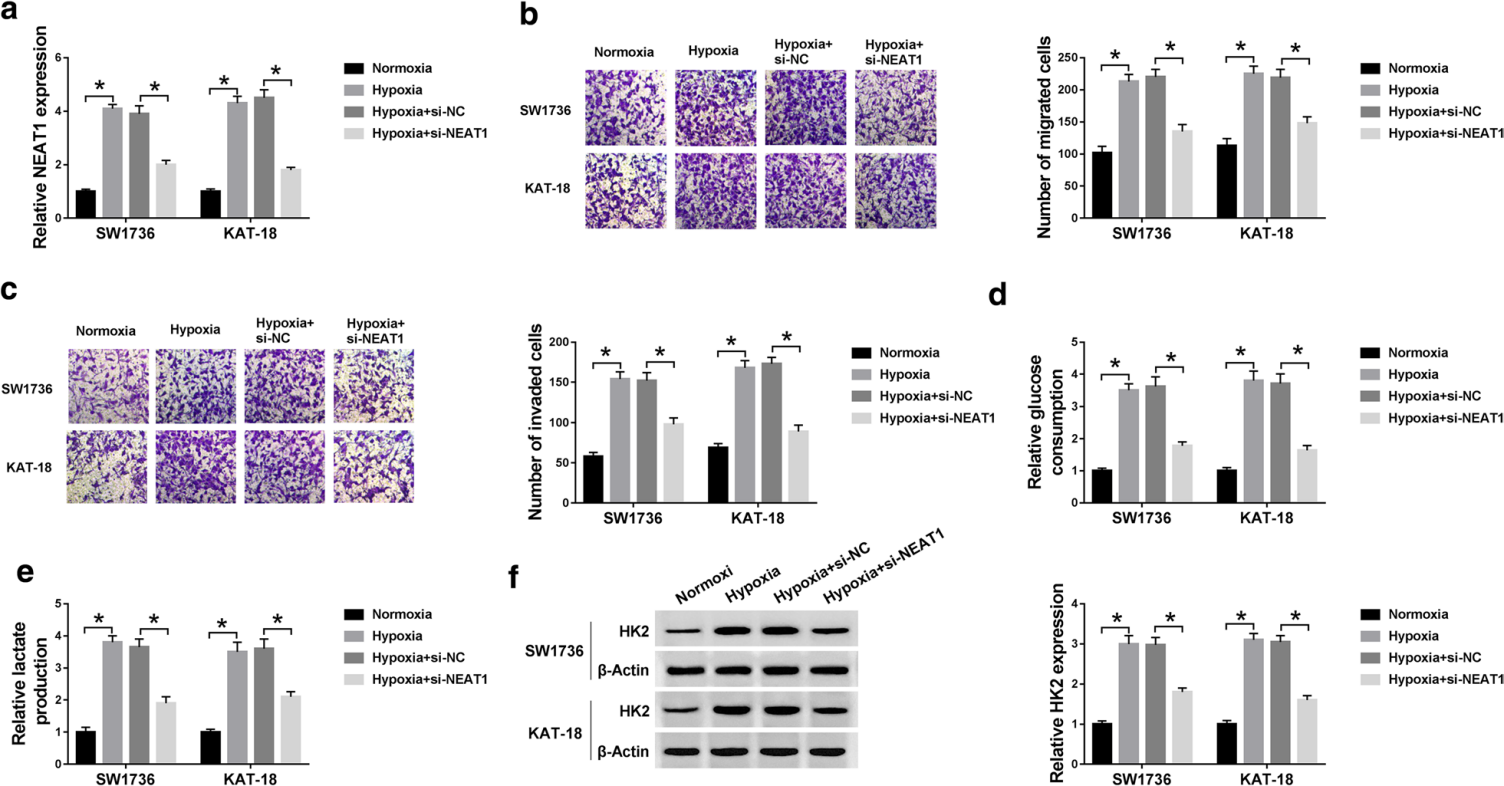
NEAT1 silencing repressed ATC cell migration, invasion and glycolysis under hypoxia. SW1736 and KAT-18 cells were exposed to 1% O_2_ or normoxia conditions for 48 h, or transfected with si-NEAT1 or si-NC before hypoxia exposure, followed by the determination of NEAT1 expression by qRT-PCR (**a**), cell migration (**b**) and invasion (**c**) by transwell assay, glucose consumption (**d**) and lactate production (**e**) using a corresponding assay kit, and HK2 expression by western blot (**f**). *P < 0.05

Given our data that hypoxia induced NEAT1 expression in both SW1736 and KAT-18 cells (Fig. 2a), we then observed cell migration, invasion and glycolysis after hypoxia treatment. Transwell results revealed that compared to the negative group, hypoxic stress led to the significant promotion in cell migration (*P* < 0.001, Fig. 2b) and invasion (*P* < 0.001, Fig. 2c). Moreover, hypoxia treatment resulted in increased glucose consumption (*P* < 0.001, Fig. 2d), lactate production (*P* < 0.001, Fig. 2e), and HK2 expression (*P* < 0.001, Fig. 2f) in the two ATC cells, suggesting the enhancement of hypoxia on cell glycolysis. To investigate the role of NEAT1 in hypoxia-induced ATC cells, we performed “phenocopy” silencing by siRNA against NEAT1 (si-NEAT1). In comparison to a negative control sequence (3.90 ± 0.31 in SW1736 cells and 4.51 ± 0.33 in KAT-18 cells), the expression of NEAT1 was prominently reduced in both SW1736 (2.01 ± 0.16) and KAT-18 (1.81 ± 0.11) cells by si-NEAT1 under hypoxia conditions (*P* < 0.001, Fig. 2a). Furthermore, the promotional effects of hypoxia on cell migration (*P* < 0.001, Fig. 2b), invasion (*P* < 0.001, Fig. 2c) and glycolysis (*P* < 0.001, Fig. 2d–f) were significantly abolished by NEAT1 knockdown in the two cells when comparing to the negative group. Taken together, these results pointed that NEAT1 silencing relieved hypoxia-induced ATC cell migration, invasion and glycolysis.

### NEAT1 acted as a molecular sponge of miR-206

**Fig. 3:**
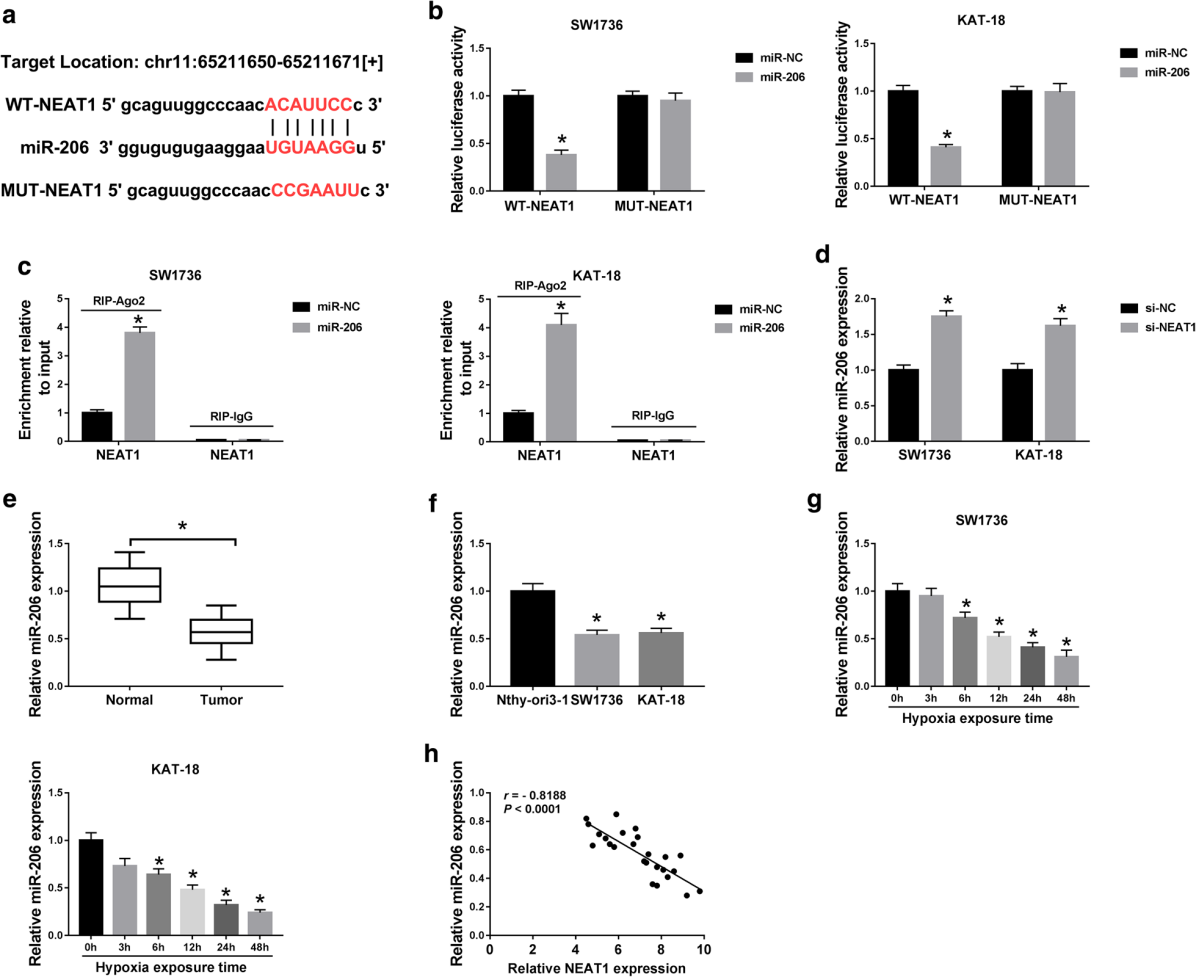
NEAT1 sequestered miR-206 by acting as a miR-206 sponge. **a** Schematic of the miR-206-binding sequence in NEAT1 and the mutant of the binding sequence. **b** Relative luciferase activity in SW1736 and KAT-18 cells cotransfected with WT-NEAT1 or MUT-NEAT1 and miR-NC mimic or miR-206 mimic. **c** NEAT1 enrichment by qRT-PCR in the RISC of SW1736 and KAT-18 cells transfected with miR-NC mimic or miR-206 mimic using anti-Ago2 or IgG antibody. **d** MiR-206 level by qRT-PCR in SW1736 and KAT-18 cells transfected with si-NC or si-NEAT1. The expression of miR-206 by qRT-PCR in 25 pairs ATC tissues and adjacent normal tissues (**e**), Nthy-ori3-1, SW1736 and KAT-18 cells (**f**), and SW1736 and KAT-18 cells exposed to 1% O_2_ for various time points (0, 3, 6, 12, 24 and 48 h) (**g**). **h** The correlation between NEAT1 level and miR-206 expression in ATC tissues using the Spearman test. *P < 0.05

LncRNAs are widely accepted to act as sponges of specific miRNAs and regulate their function. Thus, to further understand the role of NEAT1, we carried out a detailed analysis for its targeted miRNAs using the starBase v.3 software. The data of bioinformatic analysis showed a putative complementary sequence for miR-206 in human NEAT1 (Fig. 3a). To confirm this, NEAT1 luciferase reporter was transfected into SW1736 and KAT-18 cells together with miR-206 mimic. With the wild-type reporter construct and miR-206 overexpression caused a significant reduction (62% down-regulation in SW1736 cells and 59% in KAT-18 cells) in luciferase activity (*P* < 0.001, Fig. 3b). To validate whether the miR-206-binding sequence was required for this effect, a mutant NEAT1 reporter, in which all predicted seven sites were mutated, was tested. Notably, this mutant no longer elicited such an effect (*P* > 0.05, Fig. 3b). Ago2, the core component of the RNA-induced silencing complex (RISC), plays an essential role in the mature process of miRNAs [22]. To verify the endogenous association between NEAT1 and miR-206, RIP experiments were performed using anti-Ago2 antibody in miR-206-overexpressing ATC cells. In comparison to the negative control, miR-206 overexpression led to a about fourfold elevation in NEAT1 enrichment when anti-Ago2 antibody was used (*P* < 0.001, Fig. 3c). Then, we determined whether the miR-206-binding sites were functional. As expected, miR-206 expression was significantly increased (1.8-fold increase in SW1736 cells and 1.6-fold in KAT-18 cells) by NEAT1 silencing in the two cells (*P* < 0.001, Fig. 3d). Our data also demonstrated that in contrast to their counterparts, miR-206 level was markedly down-regulated in ATC tissues (about 48% reduction, *P* < 0.001, Fig. 3e) and cells (46% reduction in SW1736 cells and 44% in KAT-18 cells, *P* < 0.001, Fig. 3f), and hypoxic stress time-dependently inhibited miR-206 expression in both SW1736 and KAT-18 cells (*P* < 0.001, Fig. 3g). Additionally, an inverse correlation between NEAT1 level and miR-206 expression was found in ATC tissues (Fig. 3h). These data together strongly established that NEAT1 sequestered miR-206 by acting as a miR-206 sponge.

### NEAT1 sequestered miR-599 by acting as a miR-599 sponge

**Fig. 4:**
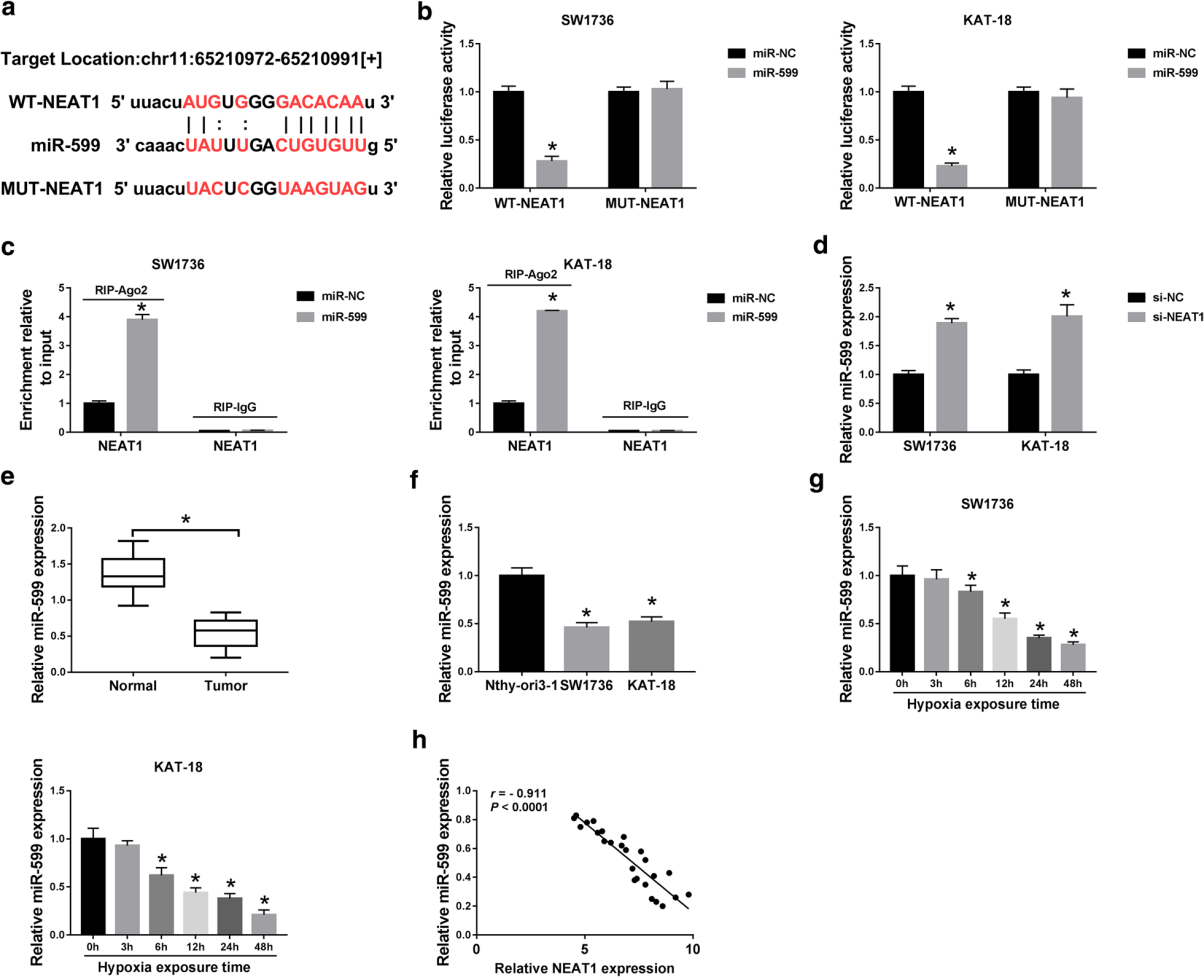
NEAT1 acted as a miR-599 sponge. **a** Schematic of the target sequence for miR-599 in NEAT1 and mutated miR-599-binding sequence. **b** The luciferase activity in SW1736 and KAT-18 cells cotransfected with WT-NEAT1′ or MUT-NEAT1′ and miR-NC mimic or miR-599 mimic. **c** NEAT1 enrichment in the RISC of SW1736 and KAT-18 cells transfected with miR-NC mimic or miR-599 mimic using anti-Ago2 or IgG antibody. The expression of miR-599 by qRT-PCR in SW1736 and KAT-18 cells transfected with si-NC or si-NEAT1 (**d**), in 25 pairs ATC tissues and adjacent normal tissues (**e**), Nthy-ori3-1, SW1736 and KAT-18 cells (**f**), the two ATC cells exposed to 1% O_2_ for various time points (0, 3, 6, 12, 24 and 48 h) (**g**). **h** Spearman test for the correlation between NEAT1 level and miR-599 expression in ATC tissues. *P < 0.05

Using the starBase v.3 software, the predicted data also revealed that NEAT1 harbored a putative target sequence for miR-599 (Fig. 4a). To verify this, we performed dual-luciferase reporter and RIP assays. Cotransfection of NEAT1 wild-type reporter and miR-599 mimic into the two ATC cells produced lower luciferase activity (72% down-regulation in SW1736 cells and 77% in KAT-18 cells) than in cells cotransfected with miR-NC mimic (*P* < 0.001), while this effect was significantly abrogated by the mutant of the target sequence (*P* > 0.05, Fig. 4b). RIP experiments showed that miR-599 overexpression triggered a remarkable up-regulation in NEAT1 enrichment (3.9-fold increase in SW1736 cells and 4.2-fold in KAT-18 cells) using anti-Ago2 antibody in the two cells (*P* < 0.001, Fig. 4c). Moreover, NEAT1 knockdown resulted in increased miR-599 expression (1.89-fold in SW1736 cells and 2.01-fold in KAT-18 cells) compared to the negative control (*P* < 0.001, Fig. 4d). In line with miR-206 level, qRT-PCR results also described that miR-599 expression was significantly reduced in ATC tissues (about 60% down-regulation, *P* < 0.001, Fig. 4e) and cells (54% down-regulation in SW1736 cells and 48% in KAT-18 cells, *P* < 0.001, Fig. 4F), and miR-599 level was highly repressed by hypoxia in a time-dependent manner (*P* < 0.001, Fig. 4g). Besides, we discovered that NEAT1 expression was inversely correlated with miR-599 level in ATC tissues (Fig. 4h). All these results pointed out the role of NEAT1 as a miR-599 sponge to sequester miR-599.

### MiR-206 or miR-599 mediated the repressive effects of NEAT1 knockdown on ATC cell migration, invasion and glycolysis under hypoxia

**Fig. 5:**
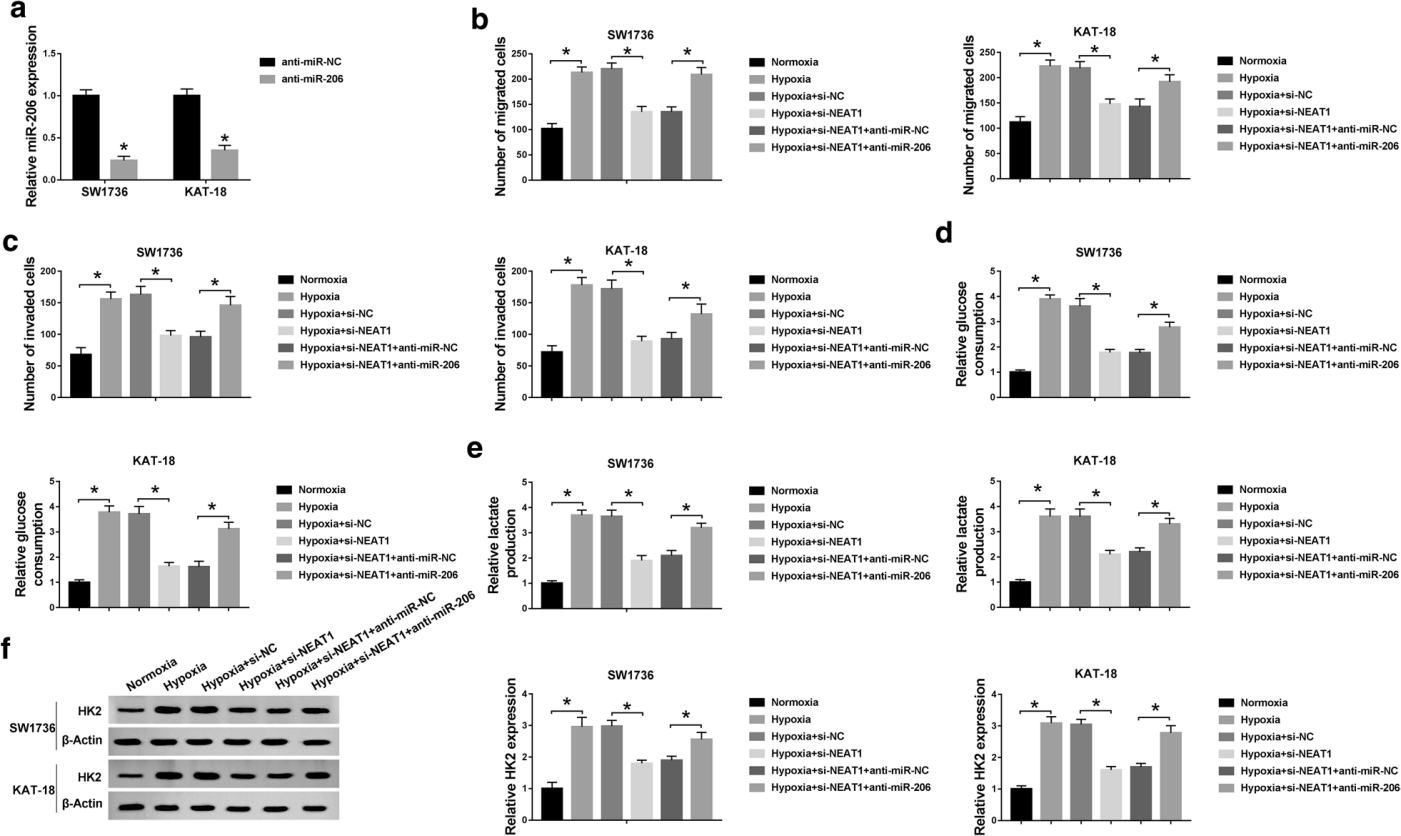
NEAT1 knockdown hampered ATC cell migration, invasion and glycolysis under hypoxia by miR-206. **a** The expression of miR-206 by qRT-PCR in SW1736 and KAT-18 cells transfected with anti-miR-NC or anti-miR-206. SW1736 and KAT-18 cells were introduced with si-NC, si-NEAT1, si-NEAT1 + anti-miR-NC or si-NEAT1 + anti-miR-206 before hypoxia exposure, followed by the detection of cell migration (**b**) and invasion (**c**) by transwell assay, glucose consumption (**d**) and lactate production (**e**) using a corresponding assay kit, and HK2 expression by western blot (**f**). *P < 0.05

To explore whether NEAT1 deficiency exerting suppressive role in ATC cell progression under hypoxia was mediated by miR-206, si-NEAT1 and anti-miR-206 were cotransfected into SW1736 and KAT-18 cells before hypoxia exposure. As shown by qRT-PCR, transfection of anti-miR-206 significantly inhibited the expression of miR-206 in the two cells (77% down-regulation in SW1736 cells and 57% in KAT-18 cells) compared with the negative group (*P* < 0.001, Fig. 5a), indicating a high transfection efficiency. Subsequent results revealed that NEAT1 depletion-mediated migration (*P* < 0.001, Fig. 5b) and invasion (*P* < 0.001, Fig. 5c) suppression were prominently abolished by the contransfection of anti-miR-206 in the two cells under hypoxic conditions. Moreover, si-NEAT1-mediated anti-glycolysis function was dramatically reversed by miR-206 silencing (*P* < 0.001, Fig. 5d–f). These data together suggested that the repressive effects of NEAT1 knockdown on ATC cell migration, invasion and glycolysis were mediated by miR-206.

**Fig. 6:**
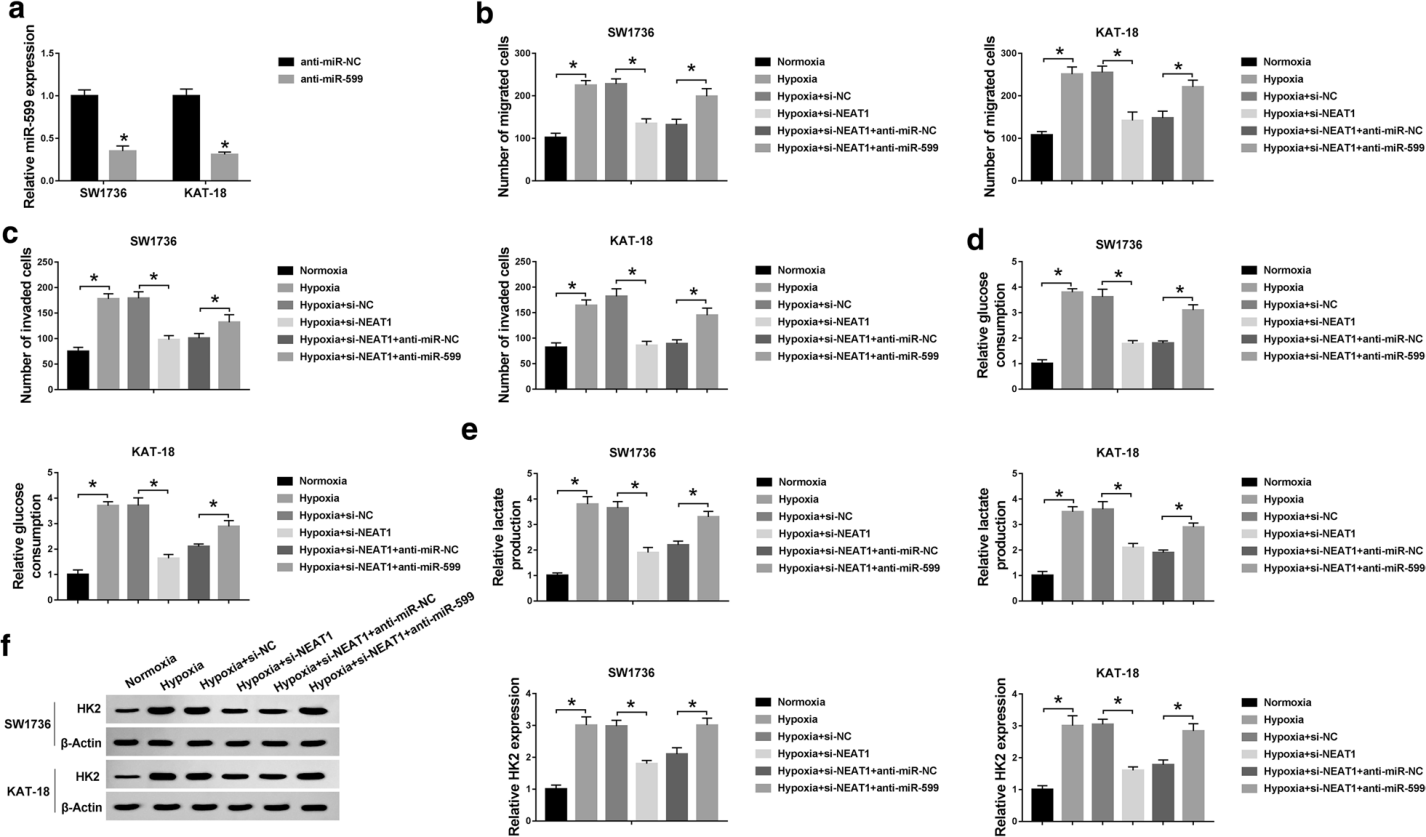
NEAT1 knockdown weakened ATC cell migration, invasion and glycolysis under hypoxia by miR-599. **a** MiR-599 level in SW1736 and KAT-18 cells transfected with anti-miR-NC or anti-miR-599. Cell migration (**b**), invasion (**c**), glucose consumption (**d**), lactate production (**e**) and HK2 expression (**f**) in SW1736 and KAT-18 cells transfected with si-NC, si-NEAT1, si-NEAT1 + anti-miR-NC or si-NEAT1 + anti-miR-599 before hypoxia exposure. *P < 0.05

Similarly, to provide further mechanistic insight into the link between NEAT1 and miR-599 on ATC cell progression under hypoxia, si-NEAT1 and anti-miR-599 were cotransfected into the two ATC cells. As shown in Fig. 6a, miR-599 level was significantly reduced by anti-miR-599 transfection (65% reduction in SW1736 cells and 69% in KAT-18 cells) compared to anti-miR-NC control (*P* < 0.001, Fig. 6a). These results revealed that in contrast to a corresponding negative group, si-NEAT1-mediated anti-migration (*P* < 0.001, Fig. 6b), anti-invasion (*P* < 0.001, Fig. 6c) and anti-glycolysis (*P* < 0.001, Fig. 6d–f) effects were remarkably abrogated by miR-599 knockdown in the two ATC cells under hypoxia. These data together implied that NEAT1 knockdown suppressed ATC cell migration, invasion and glycolysis by up-regulating miR-599.

### Knockdown of NEAT1 suppressed tumor growth in vivo

**Fig. 7:**
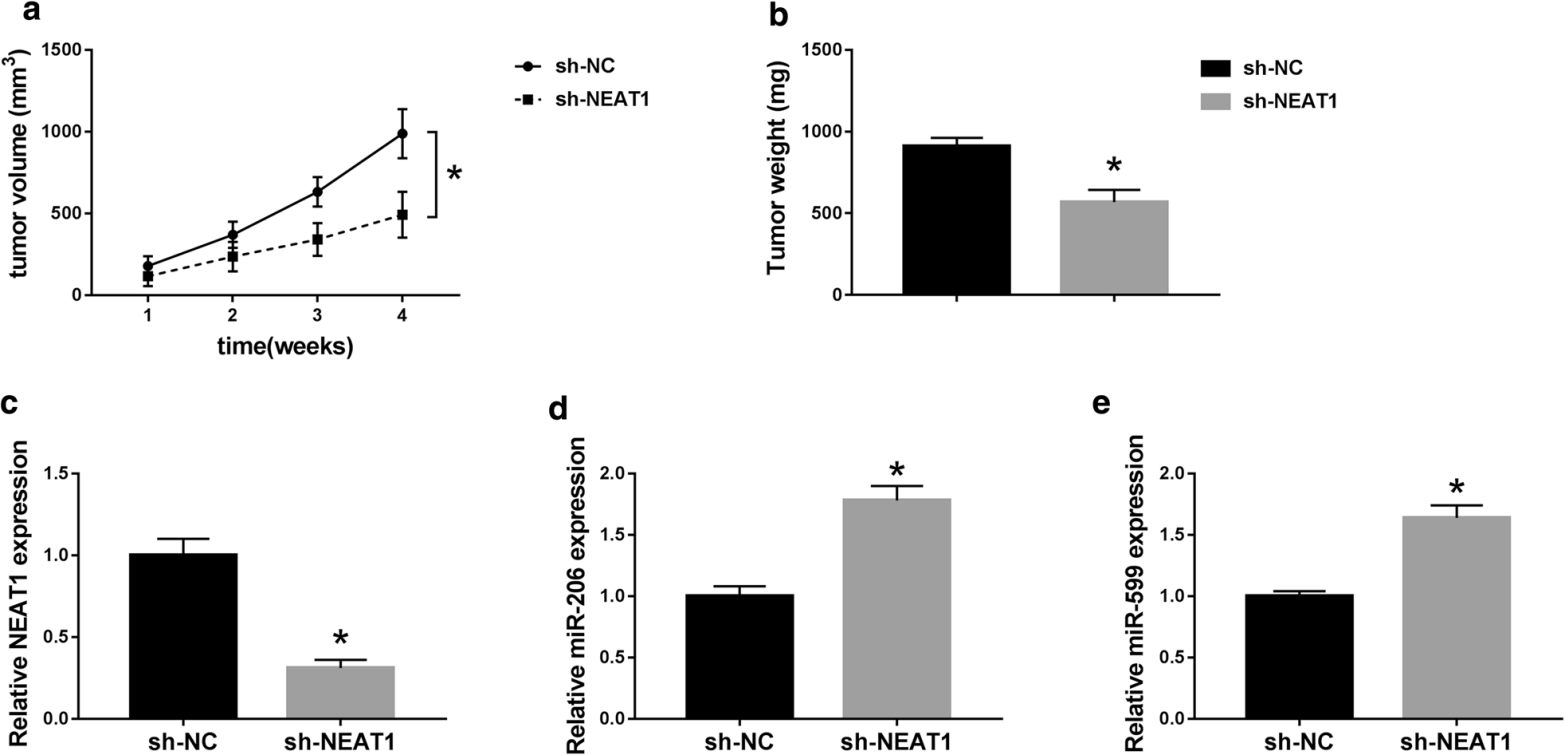
NEAT1 knockdown repressed tumor growth in vivo. About 5.0 × 10^6^ SW1736 cells stably transduced with sh-NC or sh-NEAT1 were subcutaneously implanted into nude mice (n = 9). After 4 weeks implantation, all mice were sacrificed and tumor tissues were removed. **a** After 1 week infection, tumor volume measurement began and was performed every week. **b** Tumor weight was determined. qRT-PCR for NEAT1 (**c**), miR-206 (**d**) and miR-599 (**e**) expression in xenograft tissues. *P < 0.05

Given the present in vitro findings, we further evaluated the anti-cancer role of NEAT1 knockdown in vivo. SW1736 cells stably transduced with sh-NC or sh-NEAT1 were subcutaneously implanted into nude mice to construct xenograft model. These results revealed that transduction of sh-NEAT1 significantly repressed tumor growth, as presented by the decrease in tumor volume (*P* < 0.001, Fig. 7a) and weight (*P* < 0.001, Fig. 7b). qRT-PCR data showed that NEAT1 expression was lower in sh-NEAT1 group (about 69% reduction) than that in sh-NC group (*P* < 0.001, Fig. 7c). Moreover, NEAT1 depletion resulted in increased expression of miR-206 (1.78-fold increase, *P* < 0.001, Fig. 7d) and miR-599 (1.64-fold, *P* < 0.001, Fig. 7e) in tumor tissues. Together, these results indicated that NEAT1 knockdown weakened tumor growth in vivo.

## Discussion

Hypoxia is a common characteristic of many types of solid tumors and contributes to an aggressive phenotype [2]. It is now becoming apparent that lncRNAs are modulated by hypoxia during tumorigenesis and tumor progression [23]. Among these ATC-related lncRNAs, NEAT1 is a critical lncRNA in response to hypoxia [24]. In the current study, for the first time, we illuminated that the silencing of NEAT1 repressed migration, invasion, and glycolysis in ATC cells under hypoxia possibly by the regulation of miR-206 and miR-599.

In cancer cells, hypoxia leads to the up-regulation of hypoxia-inducible factor (HIF), which directly alter NEAT1 expression in transcriptional regulation [24, 25]. Similarly, our data validated that hypoxia induced NEAT1 expression in ATC cells in a time-dependent manner. Considering the enhancement of NEAT1 on ATC chemoresistance [17], we assumed the essential involvement of NEAT1 in ATC progression under hypoxia, and our data for the first time uncovered that NEAT1 depletion weakened ATC cell migration and invasion under hypoxia. Hypoxia is widely accepted to trigger the reprogramming of cancer metabolism, leading to increased glucose consumption and lactate production [26]. Moreover, a high HK2 level in tumor cells often indicates the enhanced glycolytic phenomenon [27]. In the present study, we were first to validate that NEAT1 knockdown retarded the glycolysis in ATC cells after hypoxia exposure. Additionally, the xenograft model assays demonstrated that NEAT1 knockdown suppressed tumor growth in vivo. Zheng et al. underscored that hypoxia induced NEAT1 expression in hepatocellular carcinoma (HCC), and NEAT1 silencing repressed HCC cell migration and invasion under hypoxia [28]. Jiang et al. highlighted that NEAT1 accelerated hypoxia-induced renal tubular epithelial apoptosis via down-regulating miR-27a-3p [29].

LncRNAs are widely acknowledged to exert their biological activities via acting as miRNAs sponges [18]. Herein, we verified that NEAT1 sequestered miR-206 and miR-599 through directly binding to them. MiR-206 and miR-599 have been established as tumor suppressors in multiple types of human malignancies [30–33]. Moreover, Wang et al. illuminated that miR-206 level was reduced in thyroid cancer tissues and cells, and a high miR-206 expression inhibited this disease progression through targeting Ras-related protein Rap-1b [34]. Zhang et al. reported that miR-206 was down-regulated in metastatic ATC tissues compared with primary tumors, and its overexpression hampered ATC cell invasion and metastasis through targeting myocardin related transcription factor A [20]. Bi and colleagues uncovered that the elevated expression of miR-599 hindered ATC cell migration and invasion by targeting T-cell intracellular antigen [21]. In the current study, for the first time, we highlighted that miR-206 or miR-599 mediated the repressive effects of NEAT1 knockdown on ATC cell migration, invasion, and glycolysis under hypoxia. Previous studies had reported that miR-206 repressed ATC metastasis by targeting MRTF-A [20], and miR-599 functioned as a tumor suppressor in ATC through targeting T-cell intracellular antigen (TIA1) [21]. Thus, more investigations about the relationship between MRTF-A and the NEAT1/miR-206 axis on ATC progression under hypoxia will be performed in further work. Moreover, whether TIA1 was involved in the NEAT1/miR-599 axis-mediation regulation on ATC progression under hypoxia was absent, which is expected to be explored in further work.

In spite of various histologic subtypes by an adequate biopsy and positive PAX8 immunohistochemical staining, ATC is almost indistinguishable from any other poorly differentiated carcinoma [35]. Recently, emerging studies have suggested that many other lncRNAs, such as PTCSC3 and HCP5, were dysregulated in ATC tissues and cells, implying that they might be promising biomarkers for ATC diagnosis [11, 36]. Moreover, a group of miRNAs, including miR-19a and miR-483-3p, were reported to be involved in ATC progression, suggesting novel therapeutic targets for ATC treatment [37, 38]. This study had led to a novel identification for the NEAT1/miR-206 and NEAT1/miR-599 axis that might function as valuable diagnostic biomarkers and therapeutic targets for ATC management in further personalized medicine.

## Conclusion

In conclusion, our study indicated that the knockdown of NEAT1 suppressed the migration, invasion, and glycolysis in ATC cells under hypoxia at least partially through regulation of miR-206 and miR-599. Therefore, the NEAT1/miR-206 and NEAT1/miR-599 axis may provide new therapeutic strategies for ATC treatment.

## Data Availability

All data generated or analysed during this study are included in this published article (and its additional files).
